# A review of high-entropy materials with their unique applications

**DOI:** 10.1007/s42114-025-01275-4

**Published:** 2025-03-03

**Authors:** Juanna Ren, Vilas Y. Kumkale, Hua Hou, Vishal S. Kadam, Chaitali V. Jagtap, Prasad E. Lokhande, Habib M. Pathan, Aricson Pereira, Hanhui Lei, Terence Xiaoteng Liu

**Affiliations:** 1 College of Materials Science and Engineering, Taiyuan University of Science and Technology, Taiyuan, 030024 China; 2 Department of Physics, Savitribai Phule Pune University, Pune, 411007 India; 3 Department of Physics, Baburaoji Gholap College, Sangvi, Pune, 411027 India; 4 Departamento de Mecánica, Facultad de Ingeniería, Universidad Tecnológica Metropolitana, Santiago, Chile; 5 Engineered Multifunctional Composites (EMC) Nanotech LLC, Knoxville, TN 37996 USA; 6 Department of Mechanical and Construction Engineering, Northumbria University, Newcastle Upon Tyne, NE1 8 UK

**Keywords:** High entropy materials (HEM), Electromagnetic wave absorption, Biomedical, Rare earth, Single phase system, Advanced ceramics

## Abstract

High-entropy materials (HEMs) constitute an innovative category of advanced materials distinguished by their distinctive atomic arrangements and remarkable multifunctional attributes. This thorough overview critically analyzes the core principles, synthesis methods, and novel applications of HEMs, emphasizing their transformative potentials in electromagnetic and biological fields. This study examines how the high configurational entropy effect, lattice distortion, and slow diffusion mechanisms facilitate the stabilization of single-phase systems including numerous primary elements. Recent advancements in HEM development have demonstrated exceptional skills in electromagnetic wave absorption, attaining reflection losses of up to − 35.10 dB via nano-domain designs and synergistic dielectric-magnetic loss mechanisms. Including rare-earth elements has substantially affected magnetic ordering and transition temperatures, with La-based compounds displaying spontaneous magnetization of approximately 15.2 emu/g. In biomedical applications, innovative HEM formulations have attained improved biocompatibility with a diminished Young’s modulus (69–140 GPa) and exceptional corrosion resistance. This review provides a detailed roadmap for researchers and engineers focused on the practical application of advanced materials, through a methodical analysis of current developments in energy storage, catalysis, electromagnetic shielding, and biological applications. We emphasize the significance of composition design and processing parameters in attaining customized features for specific technological applications while recognizing key difficulties and future research avenues in this swiftly advancing sector.

## Introduction and scope of review

High-entropy materials (HEMs) have emerged as a groundbreaking category of advanced materials since its first report in 2004 by both Jien-Wei Yeh’s group in Taiwan and Brian Cantor’s group in the UK [1, 2]. These materials, distinguished by their multi-principal elements such as unique compositions, microstructure, and adjustable properties have transformed the field of materials science by contesting traditional alloy design assumptions [3–6]. Recent studies have revealed that the incorporation of rare-earth elements (Y, La, Ce, Nd) into high-entropy systems produces unique electronic band structures and magnetic ordering [7]. La and Nd were incorporated in high-entropy perovskites (La,Nd) (Cr_0.2_Mn_0.2_Fe_0.2_Co_0.2_Ni_0.2_)O_3_ and showed a significant improvement in magnetic ordering and responses. The La-based compound exhibits the highest spontaneous magnetization of ~ 15.2 emu/g at low temperatures, while Nd-based and La/Nd mixed compounds showed lower values of ~ 0.7 emu/g and ~ 0.6 emu/g, respectively, showing how different rare earth elements can be used to tune the magnetic properties [8]. The magnetic ordering temperatures (Tmo) vary systematically with composition: La-CMFCNO shows Tmo ~ 131.1 K, Nd-CMFCNO ~ 53.4 K, and the mixed La0.5Nd0.5-CMFCNO ~ 64.9 K suggesting the use of rare earth mineral to tune magnetic transition temperature. The presence of rare earth elements also influences magnetic cluster formation, with La-CMFCNO showing larger ferromagnetic clusters (~ 100 nm) compared to La0.5Nd0.5-CMFCNO (~ 60 nm), while Nd-CMFCNO exhibits spin glass behavior with clusters below 30 nm suggesting the use of rare earth mineral to control magnetic structure at nanoscale [9, 10]. This could be due to the difference in electronic configurations and ionic radii between rare earth elements. The fundamental principles of entropy engineering were developed through investigation of phase stability in multi-component metallic systems. HEAs like CuCoNiCrAlFe have shown basic entropy stabilization, but by adding refractory metals like Mo, Nb, Ta, V, and W has revolutionized the field by unlocking unprecedented thermal stability. Studies have shown that refractory metal-containing HEAs are exceptionally resistance to structural degradation at temperatures up to 1500 °C [11, 12]. High entropy materials containing rare earth elements demonstrate improved stability against oxidation and corrosion [4, 13, 14], and the specific mechanisms and quantitative improvements require further investigation. Multiple authors suggest that when the alloy’s element count rises, the entropic contribution rises as well, surpassing the enthalpic contribution and stabilizing the solid solution and having remarkable proportions, yielding distinctive characteristics that usually exceed conventional materials [15, 16].

The exceptional characteristics of HEMs, such as elevated strength and ductility [16–18], hardness, thermal stability [19–22], outstanding fatigue [23], fracture resistance [15], superconductivity [24–26], as well as resistance to corrosion, oxidation [4, 6, 13, 14], and radiation [27, 28], to their complex arrangement of numerous components at the microstructure and atomic level, have garnered considerable interest from the scientific and engineering sectors. Rare metals like Ta and Nb are particularly important in stabilizing refractory HEAs for use in high-temperature aerospace components.

Comprehensive studies have been undertaken to examine the microstructures, characteristics, and prospective uses of HEMs [4, 29, 30]. These investigations have resulted in the creation of multiple high-entropy materials (HEMs), encompassing high-entropy alloys (HEAs), high-entropy ceramics (HECs) [31, 32], high-entropy oxides (HEOs) [33], and high-entropy polymers (HEPs) [21, 34–37]. The distinctive characteristics of HEMs have a significant configurational entropy effect that helps the development of single-phase solid solutions comprising several primary constituents. This action results in various unique phenomena, including lattice distortion, sluggish diffusion, and the cocktail effect, which enhance the properties of HEMs [38–42].

HEM mechanical properties lead to multi-industry applications that include microwave absorption and electromagnetic interference (EMI) shielding [43–45]. Microwave absorption is required in multiple domains, such as telecommunications, radar systems, and stealth technologies [46–49]. The intrinsic structural complexity of high-entropy materials, including high-entropy oxides and high-entropy ceramics, allows for significant dielectric and magnetic losses, which are crucial for effective microwave absorption. The adjustable composition of HEMs facilitates the optimization of electromagnetic properties, resulting in improved absorption performance over a wide frequency spectrum. Electromagnetic interference shielding is gaining significance due to the widespread use of electronic gadgets and advancements in wireless communication systems. HEMs, with their improved electrical conductivity and magnetic permeability, provide excellent EMI shielding performance. Their ability to weaken electromagnetic waves using dielectric loss mechanisms makes them ideal for protecting sensitive electronics from electromagnetic interference. Recent studies show that high-entropy spinel ferrites and other HEM-based composites can achieve significant microwave absorption and electromagnetic interference shielding effectiveness by combining the synergistic capabilities of many elements. These materials have a wide absorption bandwidth and lightweight qualities, making them ideal for aerospace and defense applications. HEMs’ versatility has made them useful in a variety of fields, including biomedical engineering [50–52], catalysis [42, 53–55], high-temperature applications in aerospace [7, 9], and electrode materials for energy storage [13, 56–60].

Over the last two decades, research on high-entropy materials (HEMs) has grown beyond metallic alloys to cover a diverse range of material systems. High-entropy oxides, carbides, borides, and metal–organic frameworks have all been created, each with distinct features and possible applications. The variety in composition and structure has created new opportunities for material design and engineering. Recent advancements in synthesis technologies have significantly increased the possibilities for HEM design and manufacture. Advanced techniques such as additive manufacturing, thin film deposition, and novel chemical synthesis processes have facilitated the development of HEMs with tailored compositions and structures, allowing for property enhancement and application-specific design [32, 51, 61–66].

This study provides a thorough examination of novel methodologies and developing applications in the synthesis of high-entropy materials. It also investigates the theoretical underpinnings of high-entropy materials (HEMs), categorizes different types of HEMs, analyzes diverse synthesis techniques, and focuses on future applications especially in new functional features such as electromagnetic interference shielding and microwave absorption capabilities, which are becoming more essential in sophisticated technological applications. Furthermore, it investigates the most recent advances in synthesis procedures, such as sophisticated manufacturing processes and innovative chemical approaches, that allow for exact control over composition and structure. The complete review includes traditional structural material uses as well as emergent sectors including energy storage, catalysis, and biomedical engineering. This systematic approach seeks to give researchers and engineers with a comprehensive overview of the current status of HEM research, emphasizing both fundamental features and practical applications, as well as identifying interesting future avenues in this quickly growing field.

## Theory of high-entropy materials

The core principle of high-entropy materials is the optimization of configurational entropy. This concept is founded on the Boltzmann hypothesis [1], which associates entropy with the quantity of potential microscopic configurations of a system. In the realm of HEMs, the configuration entropy (ΔSconf) is expressed as [67]:

$$\Delta Sconf = -R \Sigma xi ln xi$$

where *R* denotes the gas constant, and xi represents the mole fraction of the *i*th component.

For a system with *n* equimolar components, this equation reduces to:

$$\Delta Sconf = R ln n$$

As the quantity of components escalates, the configurational entropy rises, resulting in a decrease in the Gibbs free energy of the system [7]. This entropy effect can stabilize solid solutions in preference to intermetallic complexes or phase-separated states [68].

The high-entropy effect is not the sole determinant affecting the creation and stability of HEMs. Additional critical factors encompass variation in atomic dimensions, enthalpy of amalgamation, concentration of valence electrons, and difference in electronegativity.

Recent research has yielded enhanced understanding of these impacts. Research on lattice distortion has demonstrated its considerable influence on the mechanical characteristics and diffusion behavior in HEMs [39–41, 68–71]. The sluggish diffusion effect, once considered universal in HEMs, has been demonstrated to be dependent on composition, with certain systems displaying “anti-sluggish” diffusion behavior [41].

The thermodynamic stability of HEMs has been extensively studied. Computational techniques, including CALPHAD (CALculation of PHAse Diagrams) and first-principles calculations, have been utilized to forecast phase stability and inform alloy design [72]. CALPHAD analyze complicated multi-component phase diagrams and predicting phase formation trends for over 130,000 different multicomponent (with 3–6 elements) alloys [73]. These methodologies have resulted in the formulation of novel HEM compositions exhibiting improved characteristics and stability.

Comprehending the interplay between composition, structure, and characteristics in HEMs presents a considerable difficulty owing to the extensive compositional space and intricate interactions among many parts. Machine learning and high-throughput experimental methodologies are emerging as potent instruments for managing this complexity and expediting the identification of novel HEMs with customized attributes [74–77].

The aforementioned factors, in conjunction with the high-entropy impact, lead to the four “core effects” of HEMs [15, 78]: High-entropy effect that promotes the development of solid solution phases and diminishes the propensity for ordering and segregation.

Significant lattice distortion arises from the atomic size discrepancy of the constituent elements, resulting in solid solution strengthening. Impeded diffusion results from heterogeneous local atomic environments, influencing phase transitions and microstructural integrity. And cocktail effect with synergistic amalgamation of attributes results from the varied constituent elements. Comprehending these theoretical principles is essential for the design and synthesis of novel HEMs with customized features for particular applications.

## Classification of high entropy materials

### Alloys

**Fig. 1:**
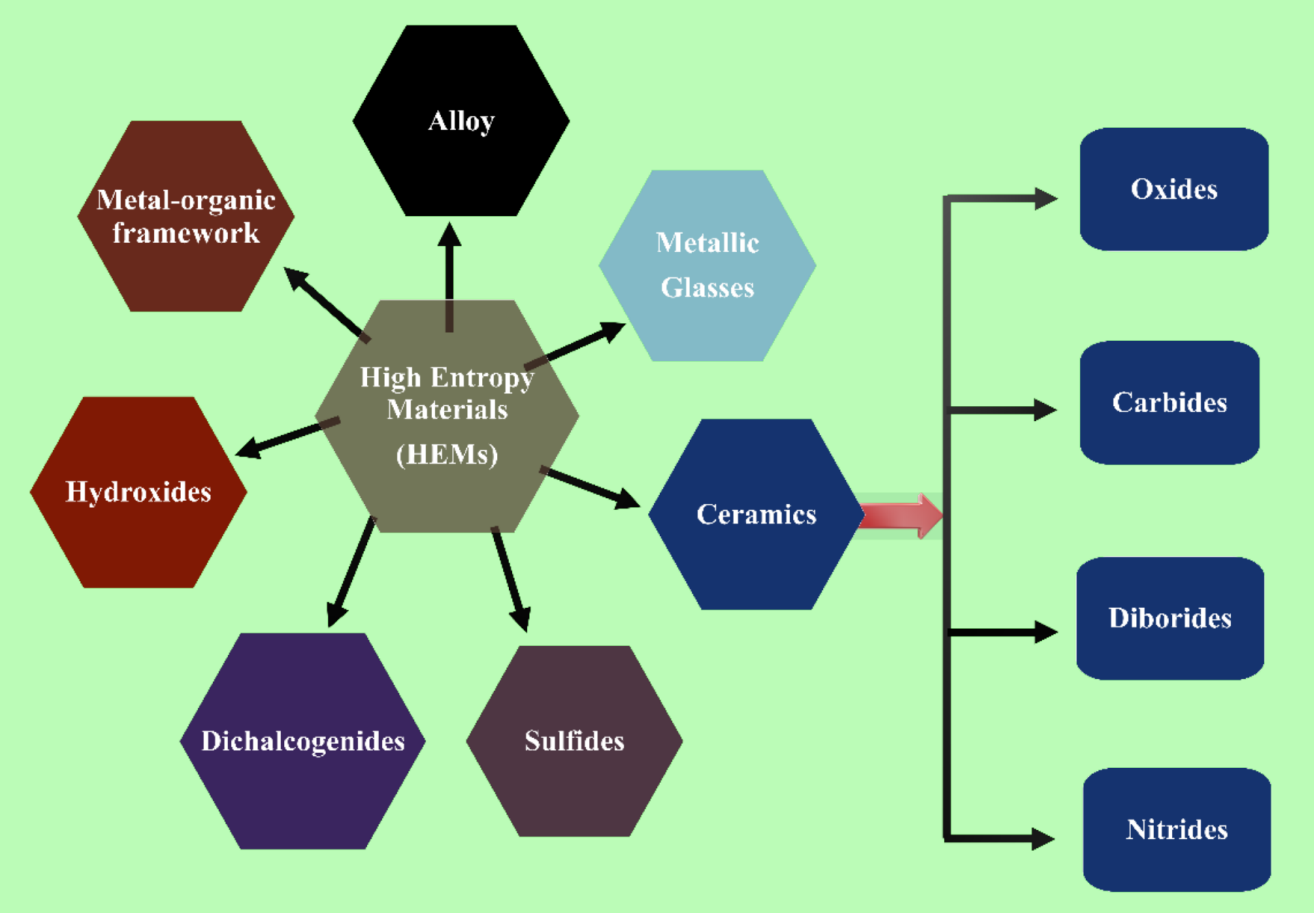
Classification of HEM alloys

To effectively control the influencing thermodynamic or kinetic parameters and create HEMs with a single solid solution phase and homogenous element distribution, several physical and chemical production methods are available. To regulate the size, phase structure, and composition distribution of high-entropy nanomaterials, the synthesis method and circumstances play a more important role [79]. HEM materials represent materials in which the phase stabilization process is driven by high configurational entropy. For intermetallic and multiphase microstructures, a disordered solid solution having a single-phase crystal structure stabilizes when mixing entropy is high [29, 80]. As shown in Fig. 1, a comprehensive classification of HEMs is based on their compositional design and entropy engineering principles.

Within the class of HEMs, HEAs are the first materials to be found. Extensive studies on HEAs over the past 10 years, since their development in 2004, have produced several intriguing outcomes, including increased hardness and strength compared to conventional materials [81]. The theory of entropy engineering was first introduced through the stability of various metal phases. CuCoNiCrAlFe and CuCoNiCrAlFeMoTiVZr HEAs were discovered by combining many (five or more) elemental species in an equiatomic or nearly equiatomic ratio into a single solid solution [1]. According to the authors, when the alloy’s element count rises, the entropic contribution rises as well, surpassing the enthalpic contribution and stabilizing the solid solution [1, 15]. Similar to the components present or completely distinct, HEAs can display a variety of novel occurrences and features. Conventional alloys consist of one or very few components, with the addition of small amounts of alloying to achieve higher qualities, such as iron in steels or nickel in superalloys [35, 82]. In contrast, HEAs like FeMnNiCoCr [16], VNbMoTaW [7], NbCrMoTaTiZr [7, 83], TaNbHfZrTiMo [29], YGdTbDyLu, and GdTbDbTmLu [72] have remarkable properties like high thermal and phase stability, high hardness, high strength, and excellent wear, corrosion, and fatigue resistance due to their complex, arbitrary arrangement of numerous components, and unique chemical interaction at the atomic level.

### Metallic glasses

Metal-based alloys are referred to be metallic glass when they are rapidly cooled from a liquid state while maintaining an amorphous liquid structure [72]. The creation of high-entropy metallic glasses (HEMGs) offers several options in this context. It has been discovered that HEMGs can exhibit superiorities due to their long-ranged disordered structure and high density of unsaturated atomic coordination. Although HEMGs have great potential for improving atomic configurations and catalytic performance, the objective of controlling functional atoms and balancing their synergistic effects to obtain superior functional characteristics remains a research challenge [41].

### Ceramics

The concept of HECs, a relatively new subject of study, was developed long before its name was determined. High-entropy nitride was created in 2004 following the description of HEAs, and the HEC family of materials was first referred to as high-entropy alloy oxide in 2012. Currently, there are families of high-entropy compounds such as nitrides, carbides, oxides, and borides. More recently, high-entropy silicides, borocarbides, and oxyhalides have also been identified [27, 84]. Solid solutions based on interstitial that include four or more metallic species give rise to high-entropy (HE) ceramics, which have special mechanical and physical characteristics due to entropy stabilization. Growing interest has been shown in HE ceramics since they outperform binary ceramics in terms of hardness, corrosion resistance, fracture toughness, and stability at high temperatures [85].

The Rost group developed HEOs in 2015, allowing for the mixing of at least five elements in a single oxide lattice [79]. Based on the general idea of HEMs, HEOs are a class of HECs and a field [33]. In this context, a novel class of metal oxides known as multicomponent metal oxides, also referred to as high entropy oxides (HEOs), has drawn a lot of interest from researchers because of their unique physicochemical characteristics. Due to the high configurational entropy effect, HEOs which are simple solid solutions consisting of at least five principal metal atoms in equal or nearly equal atomic ratios mostly crystallize into single-phase crystal structures like perovskite, spinel, rock-salt, fluorite, or single-phase rock-salt rather than adopting multiple intermetallic phases. In the context of single-phase rock-salt crystal formations, for instance, metal atoms are joined together and arranged according to thermodynamic principles to produce the structure V-W—X–Y-Z-O, where V, W, X, Y, and Z stand for the major metal atom and O for oxygen. When their size is reduced to the nanoregion, they gain unique physicochemical qualities such as superior electrical and magnetic properties, high mechanical and thermal stability, and resistance to corrosion and oxidation [4, 13, 14].

A variety of techniques exist for producing HEO nanoparticles, including spray pyrolysis, coprecipitation, hydrothermal method, sol–gel autocombustion method, wet-chemical approach, pulse-laser ablation, and mechanical milling. The easiest and most effective technique for large-scale synthesis is sol–gel auto combustion [78].

An exclusive class of ultra-high temperature ceramics are borides of the groups IVB and VB transition metals with boron (ZrB_2_, ReB_2_, WB_4_, CrB_4_, MnB_4_). Because of their high melting temperature, high-temperature strength, thermomechanical properties, and chemical properties, these materials are among the most desirable alternatives for a variety of applications, including cutting tools, microelectronics, and aerospace [86, 87]. They offer considerable promise for applications that need extraordinary mechanical performance and ultrahigh chemical stabilities, such as rock and mineral drillbits, tram and train wheels, wearing-resistant portions of micro-electronic systems, and spaceship protection layers.

The idea of high entropy is extended to various materials as the study progresses. Recent research publications have focused emphasis on high-entropy transition metal carbides (HETMCs) as a type of advanced ceramic material. Typically, HETMCs consist of the C element and five or more transition metals, with the C element occupying the anion position and the metal components sharing a cation position. Similar to the surprising characteristics of HEAs, HETMCs also exhibit lattice distortions, slow kinetics, high entropy effects, and synergistic effects [1, 88–90]. Because of their greater melting temperatures, carbides are another unique type of ultra-high temperature ceramic that may find use in nuclear energy and aerospace applications [86].

With the addition of elements like N, C, and B, especially high-entropy nitride (HEN), high-entropy ceramics quickly followed and gained desirable qualities [90]. Overall, the combination of HEN's properties results in significantly improved mechanical behavior, thermodynamic properties, and dynamics performance. This means that HEN has a wide range of potential applications, including diffusion barriers, corrosion resistance, supercapacitors, and thermal and environmental protection [91–93]. As special features were investigated, the multielement TMNs system proved to be of significant interest. Remarkably, by embracing the concept of entropy engineering, many research groups transcended the conventional quadruple TMNs system and created multiple HENs systems, including (AlCrMoSiTi)N, (AlMoNbSiTaTiVZr)_50_N_50_, (Ti-Hf-Zr-V-Nb)N, and (TiZrHfVNbTa)N [70, 94, 95].

Co-crystallization of two distinct organic molecules is possible in organic materials, which comprise a vast pool of carbon-based chemicals. This expands the characteristics of organic crystals, making it a viable method for the development of HEPs. When the spatial packing scheme of structural units is determined by lattice interactions and the axial rotation of structural units inside the crystalline lattice, organic co-crystals, unlike metal alloys, get their entropy from the permissible change in molecular conformation.

### Sulfides

Materials with many cations and sulfur functioning as an anion are known as HESs. Cu–S compounds are inexpensive, environmentally acceptable minerals with a diamond-like structure and poor thermal conductivity. Recent advancements in HES synthesis have exhibited exceptional control over composition and characteristics [96]. The integration of various transition metal cations (including Cu, Fe, Co, Ni, Zn) into sulfide frameworks facilitates adjustable electrical, magnetic, and catalytic properties. Cu–S compounds are cost-effective, ecologically friendly minerals characterized by a diamond-like structure and low thermal conductivity.

The synthesis-property correlations in HESs have been thoroughly investigated, indicating that regulated nanoparticle synthesis may produce sizes ranging from 4 to 12 nm with homogeneous component distribution [96]. These materials are more stable than typical sulfides, thanks to the high configurational entropy impact [97].

Promising results have emerged in electrocatalysis where entropy-stabilized (CuAgZnCoMnInGa)S material has proven to be an excellent electrocatalyst for the hydrogen evolution reaction when combined with conducting carbon black, achieving a low onset overpotential of (∼80 mV) and η10 of (∼255 mV) [98]. The enhanced activity is attributed to the synergistic effects between multiple metal sites and optimized electronic structure [98]. Also, (CrMnFeCoNi)Sx nanoparticles show one of the best activities (low overpotential 295 mV at 100 mA cm^−2^ in 1 m KOH solution) and good durability (only slight polarization after 10 h by chronopotentiometry) [99]. The material maintains structural stability during extended operation, with post-catalysis characterization showing minimal surface reconstruction [99]. (CrMnFeCoNi)S_x_ and FeNiCoCrMnS_2_ exhibit superior oxygen evolution reaction (OER) activity with an exceptionally low overpotential of 199, 246, 285, and 308 mV at current densities of 10, 100, 500, and 1000 mA cm^–2^, both of these high entropy materials showing excellent performance compared with their unary, binary, ternary, and quaternary sulfide counterparts [97].

Recent research has also revealed the feasibility of combining novel synthetic methodologies, such as electrochemical approaches, to produce HES with improved catalytic characteristics [100]. The combination of numerous metal centers produces plentiful active sites and tailored electronic structures, resulting in better mass activity (756.3 mA mg⁻^1^ at η = 300 mV) [100]. This exceptional OER performance of the high entropy sulfide brings a great opportunity for desirable catalyst design for practical applications.

### Dichalcogenides

A class of layered compounds known as transition metal dichalcogenides (TMDCs) have the generic chemical formula MX_2_, where M stands for transition metals (such as Ti, Zr, Hf, V, Nb, Ta, and Cr) and X represents chalcogen elements (S, Se, and Te) [101]. These materials show strong in-plane covalent bonding and weak van der Waals interactions between layers, allowing for the development of stable 2D structures [101]. The introduction of high-entropy TMDCs has opened up new possibilities for 2D material design. Recent breakthrough research has shown that including five or more transition metals into the TMDC structure can produce stable, high-entropy 2D phases [102]. The (Mo,V,Nb,W,Ta)S₂ system differs from standard TMDCs due to its excellent stability and unique electrical properties [102]. The high configurational entropy stabilizes the mixed-metal structure while also allowing for precise control of the electronic band structure and catalytic properties. The high-entropy strategy in TMDCs provides several unique advantages, including enhanced catalytic activity through multiple active sites, improved stability under operational conditions, tunable electronic properties through composition control, and resistance to phase separation due to entropy stabilization [102]. TMDCs have demonstrated interesting uses in numerous industries because of their superior physical characteristics and diverse chemical compositions, particularly their low-dimensional feature [103]. TMDCs can be an effective approach for creating high-entropy 2D or quasi-2D materials because of their adaptable chemical compositions and structural tolerance [33]. Recent studies show that high-entropy TMDCs can achieve CO₂ reduction current densities exceeding 300 mA/cm^2^ with Faradaic efficiencies above 90%, significantly outperforming traditional TMDCs [102].

### Hydroxides

High entropy layered double hydroxides (HE-LDH) are regarded to offer the best promise for the development of noble transition metal-based OER electrocatalysts [104, 105]. HE-LDHs, a type of ionic lamellar blend, are made up of positively charged brucite-like piles that have a high capacity to intercalate anions and solvation molecules. Recent advances show tunable electronic properties through composition control [105]. For example, five transition metals (Fe, Co, Ni, Cu, Zn) into LDH structures have achieved exceptional OER performance with overpotentials as low as 230 mV at 10 mA cm⁻^2^ and long-term stability exceeding 100 h [105]. The synergistic effects between metal cations create abundant active sites and optimize electronic structures, contributing to better catalytic performance. They are widely used in a variety of fields, including polymerization, biomedical science, electrochemistry, magnetization, and environmental protection [106]. The formula [AcB Z AcB]n depicts the usual layered sequence in LDHs, where A and B stand for the layers of hydroxide anions (HO-), Z for neutral molecules (like water) and layers of other anions, and c for the layers of metal cations. HE-LDHs have shown promise in water splitting applications with high current densities, selective gas separation membranes [107], energy storage devices, and environmental remediation/carbon capture systems.

### Metal–organic framework

**Fig. 2:**
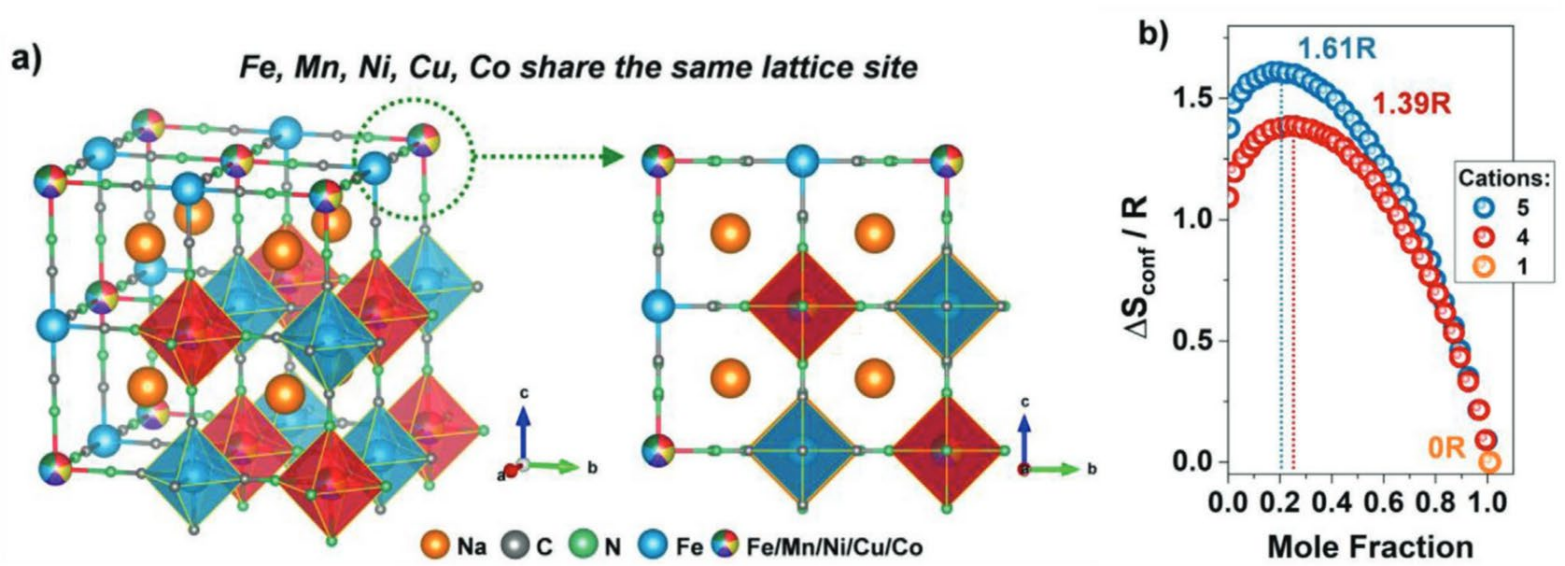
**a** The crystal structure of HE-MOF showing five different metal species sharing the nitrogen-coordinated site, showing the high-entropy design principle. The configurational entropy increases with the number of metal species as shown in (**b**), enabling enhanced stability through entropy effects [108]

High-entropy metal–organic frameworks (HE-MOFs) represent a significant advancement to the foundation work provided by Yaghi and Li in the late 1990s. MOFs are very porous materials (~ 0.13 gm/cm^3^) with high surface areas (up to 10,000 m^2^ g^1^), enormous pore volumes, and clearly defined pore size distributions that characterize these very light (B_0.13_ g cm^3^) materials. The significant class of crystalline porous materials known as metal–organic frameworks (MOF) is made up of metal ions and/or clusters and organic ligands, see Fig. 2 [108, 109]. Its notable properties include a large specific surface area, regular and controllable pore structures, and a diversity of chemical compositions and functionalities [59, 60, 110]. Recent developments show that incorporating five or more metal centers creates frameworks with high configurational entropy, leading to enhanced stability and performance [111].

**Fig. 3:**
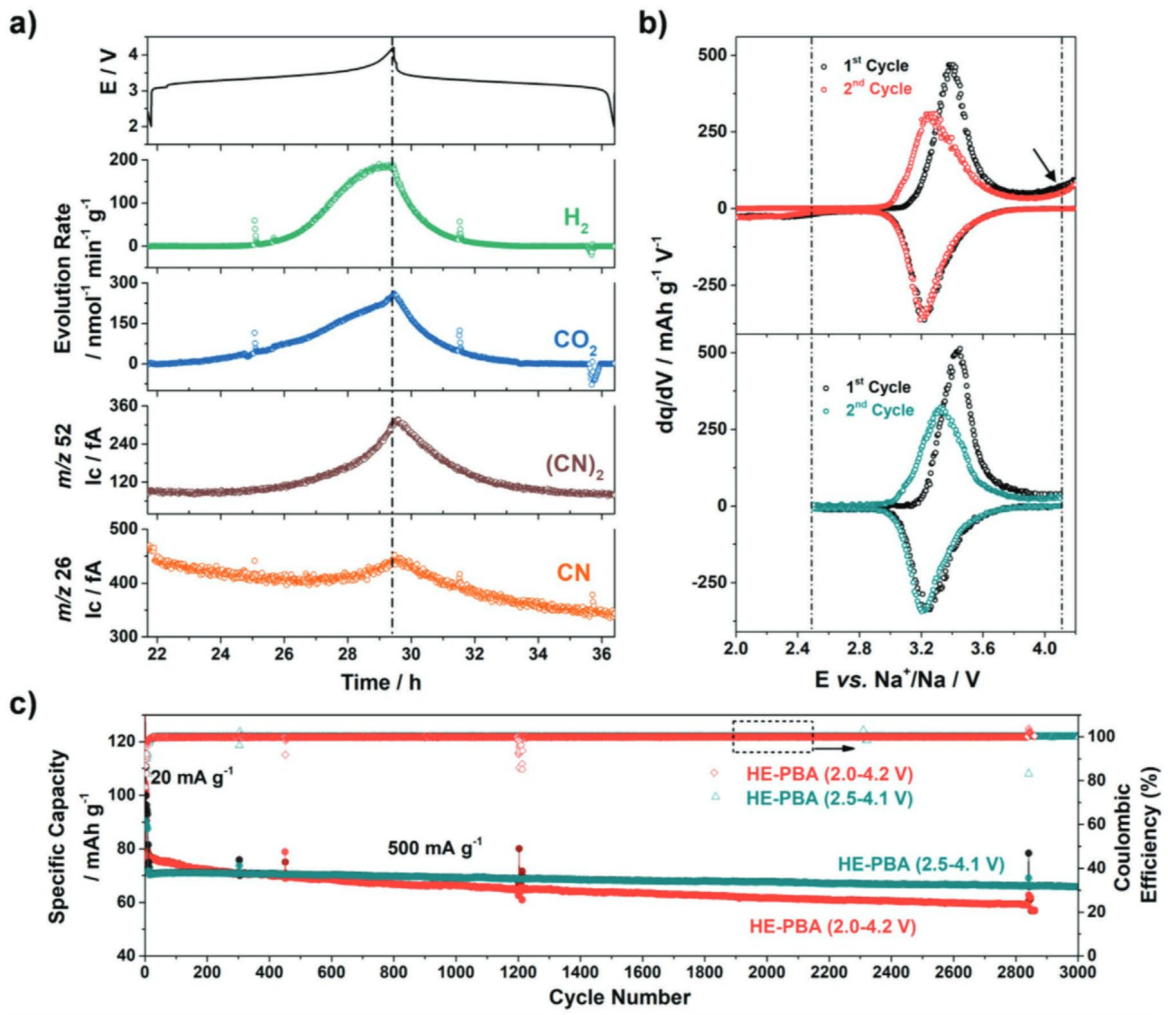
Electrochemical performance of HE-MOF. **a** Correlation between voltage profile and gas evolution rates showing stability under operating conditions. **b** Differential capacity plots comparing cycling in different voltage ranges (2.0–4.2 V vs. 2.5–4.1 V). **c** Long-term cycling performance at 0.5 A g⁻^1^ demonstrating exceptional stability over 3000 cycles [108]

Entropy-stabilized MOFs have achieved remarkable sodium storage capacity exceeding 400 mAh g⁻^1^ with superior cycling stability shown in Fig. 3 [108] as well as outstanding catalytic activity for oxygen evolution processes [112]. Incorporating various transition metals (Fe, Co, Ni, Cu, Zn) in precise ratios has allowed for more control over framework topology and pore design [113]. The synthesis of these materials has advanced dramatically, with methods spanning from ambient temperature to ultrasonic-assisted assembly [114], allowing for fine control over structure and composition.

HE-MOFs outperform typical MOFs in electrochemical water-splitting applications, with current densities exceeding 100 mA cm⁻^2^ at overpotentials around 300 mV [109]. Recent breakthrough investigations have revealed that rational design of metal node combinations can achieve ultrahigh CO₂ capture capacity of 12.3 mmol/g at 273 K [113]. MOFs are a type of porous polymeric material that is useful in many different applications, including membranes, thin-film electronics, gas separation, catalysis, and biomedical imaging. They are made up of metal ions connected by organic bridging ligands [115]. The key advantage is the incorporation of multiple metal centers which not only increases stability through entropy effects but also creates synergistic interactions, resulting in improved functional properties such as gas adsorption selectivity and catalytic activity [116]. These materials demonstrate extraordinary stability under extreme circumstances while retaining structural integrity and performance, overcoming significant shortcomings of typical MOFs [117]. The combination of entropy stabilization and precise metal ratio control allows for unparalleled tuning of framework properties and performance measures [113].

## Synthesis methods of HEM’s

**Fig. 4:**
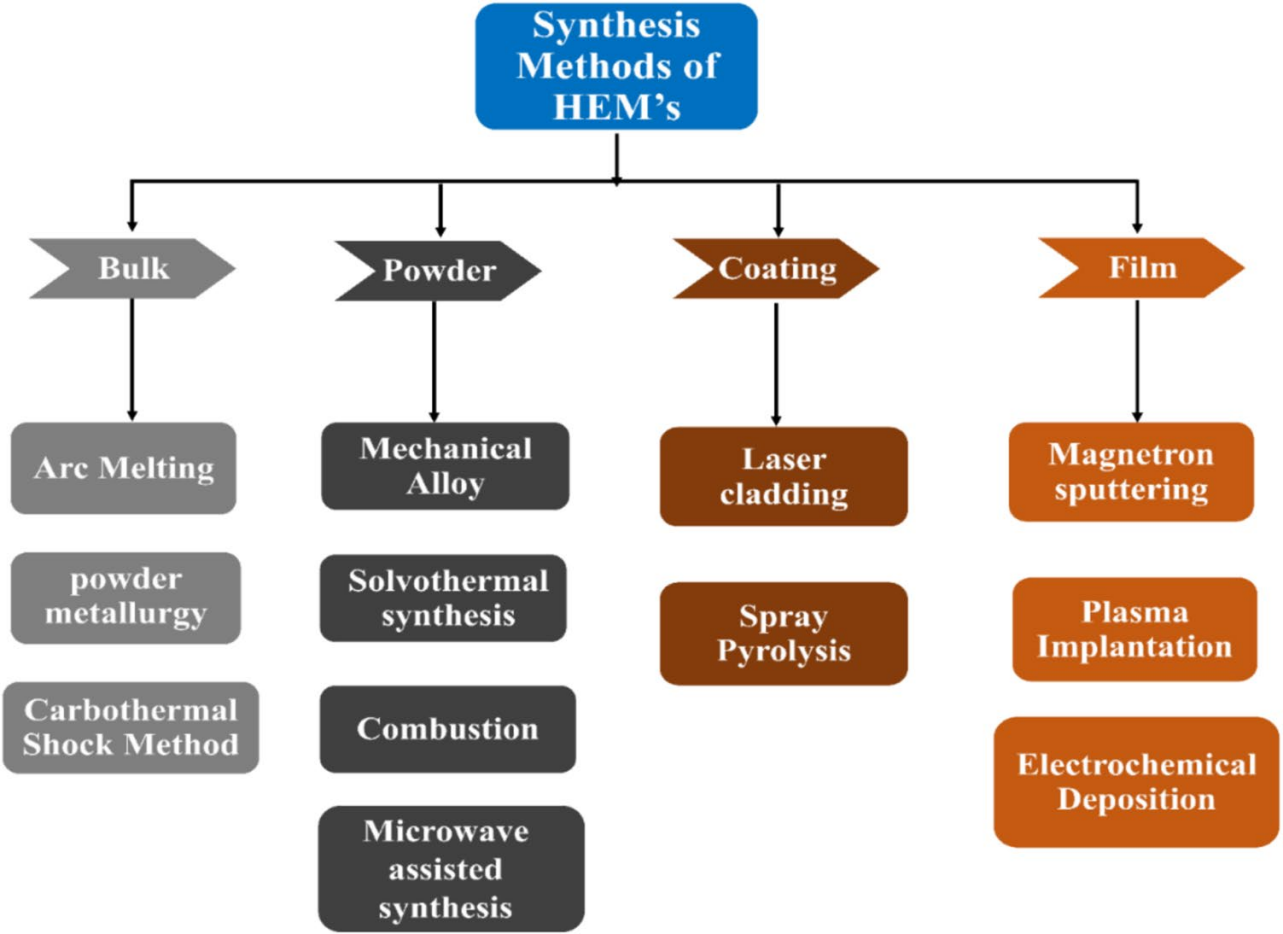
Traditional synthesis methods for HEMs

HEMs require various physical and chemical synthesis pathways that can be employed to manipulate thermodynamic factors, effectively producing high-entropy materials (HEMs) characterized by a single solid solution phase and uniformly distributed elements. The synthesis method and conditions play a pivotal role in shaping high-entropy nanomaterials, influencing factors such as size, phase structure, and composition distribution. Figure 4 shows the various types of synthesis methods of HEMs.

**Fig. 5:**
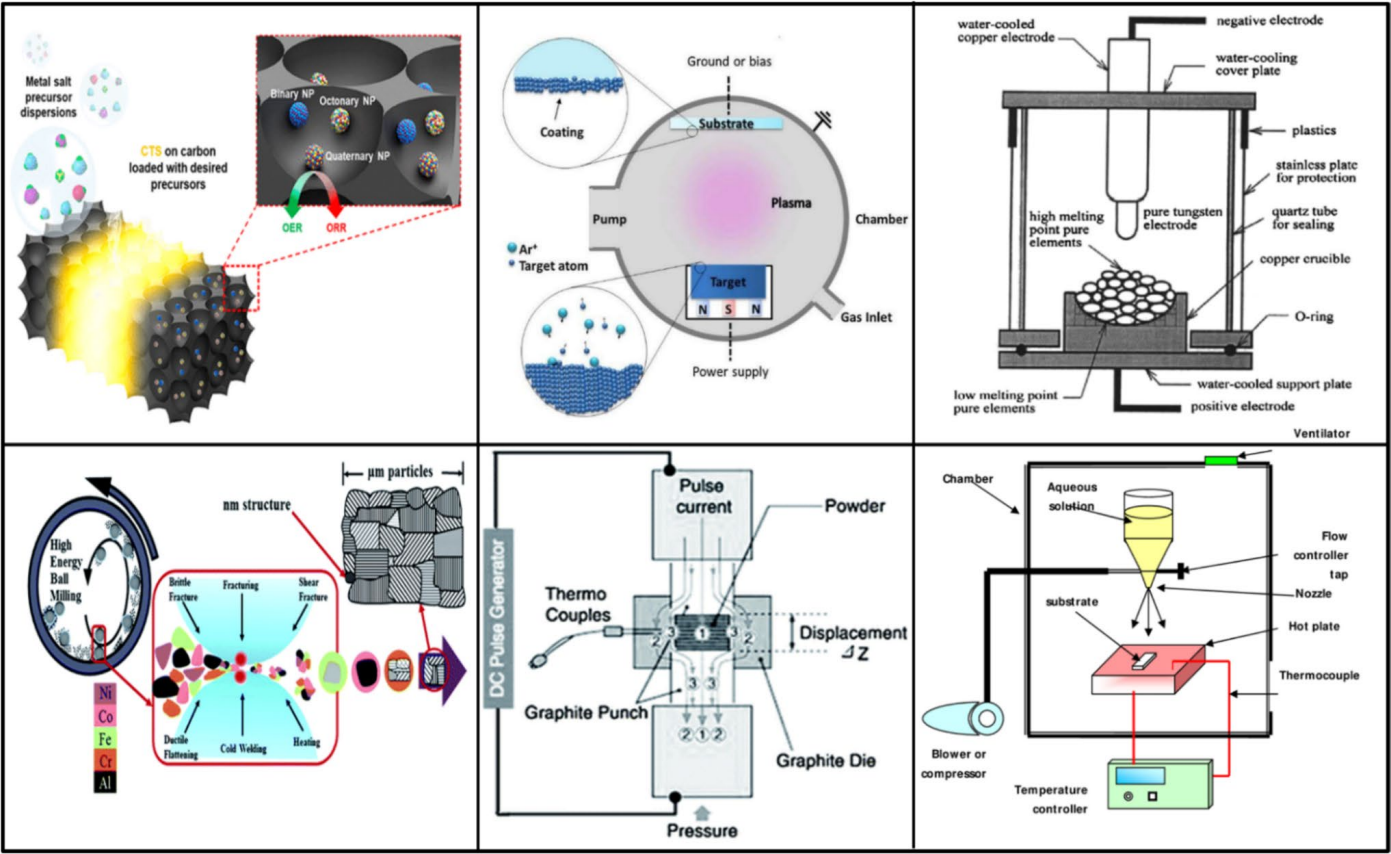
Traditional synthesis methods for HEMs. **a** Carbo-thermal shock [119] CTS synthesis method. **b** Magnetron sputtering [120]. **c** Arc melting method [30]. **d** Mechanical alloying method [121]. **e** Plasma implantation [122]. f Spray pyrolysis method [61]

HEAs have been prepared by a variety of synthetic methods, including solid-state processing, mechanical alloying, arc melting, induction melting, casting, and magnetron sputtering, see Fig. 5. These extensive synthesis processes are classified as chemical (chemical vapor deposition (CVD) and liquid phase deposition) or physical (physical vapor deposition, including vacuum sputtering, vacuum evaporation, and ion plating) synthesis routes [118]. Since the constituent elements must be uniformly and randomly dispersed at the atomic level across the crystal lattice to maximize the configurational entropy of HE materials, it is challenging to do this using conventional solid-state approaches. For the production of HE materials, many alternative methods have been documented, such as molecular precursor techniques, sol–gel synthesis, solvothermal synthesis, molecular-precursor approaches, and carbothermal synthesis. Using atoms or molecules to atomically create the high-entropy lattice rather than modifying premade lattices is a common feature of all these atom-up techniques.

### Arc melting

An electric arc is created by conducting electricity between the electrodes or between the electrodes and the metal-melting materials in the electrothermal metallurgical process known as arc melting. Direct current is used over alternating current since it is less unstable and would not disrupt melting. Both can be used to create the arc. The arc melting method is a technique used for melting and solidifying materials, typically metals or alloys, by generating an electric arc between the material and an electrode. The molten material is rapidly quenched to form a solid with a high-entropy configuration. Arc melting is a technique used for the synthesis of high entropy materials (HEMs), particularly alloys, by melting and solidifying a mixture of constituent elements. Researchers and engineers should be mindful of equipment costs, energy consumption, and potential limitations in elemental choices when employing this technique.

The refractory Hf_25_Nb_25_Nb_25_Ti_25_Zr_25_ high entropy alloy with the BCC structure was produced by Wu et al. by preparing alloy ingots by melting pure metals in an electric arc furnace and cooling the melted alloy by high-temperature treatment in a water-cooled copper mold. The best result is achieved at a temperature of 1300 °C for 6 h in an argon environment [123]. Liu et al. used arc melting to create ingots with the atomic percentage formula Fe_20_Co_20_Ni_20_Cr_20_Mn_20_. The FeCoNiCrMn high entropy alloy with a unifacially centered cubic structure is obtained by remelting the ingots four times. Grain boundary motion is governed by a solute-resistance mechanism as the alloy softens and the grains develop. At temperatures above 850 °C, the alloy’s strength follows the classical Hall–Petch relationship [124].

### Powder metallurgy

Powder metallurgy is a method utilized in the synthesis of high entropy materials (HEMs), wherein elemental powders are mechanically blended and subsequently subjected to processes like compaction and sintering. Powder metallurgy provides benefits like uniform composition and precise microstructure control. Careful consideration is needed for challenges such as elevated temperature demands, potential contamination, and equipment expenses when choosing this synthesis method. Based on the high entropy alloy made using powder metallurgy (PM), Qiu et al. studied the mechanical properties of AlCrFeNiCoCu. A measurement of 583 HV and 1341 MPa was found for the compressive strength and hardness [30]. The high entropy alloy of Al_0.6_NiFeCrCo produced by powder metallurgy was the subject of an investigation by Fu et al. into the mechanical characteristics and effects of Co on microstructure and mechanical behavior. High entropy alloys include Al_0.6_NiFeCrCo and Al_0.6_NiFeCr. 1870 MPa, 2150 MPa, 10.6%, and 570 HV were discovered to be the yield strength, compressive strength, fracture strain, and hardness, respectively [78]. FeCoCrNi high entropy alloys were invented by Lui et al. via powder metallurgy (PM). PM FeCoCrNi high entropy alloys have a high elongation of 56% and a high tensile strength of 712.5 MPa [125].

### Solvothermal synthesis

Solvothermal synthesis utilizes high temperatures and pressures with a solvent to control reaction conditions, resulting in the formation of distinct materials with specific properties. For complex compositions, solvothermal synthesis has advantages in terms of fine particle size, homogenous composition, and adaptability. However, while thinking about using this process for the synthesis of high entropy materials, the difficulties associated with high temperature, pressure, energy consumption, and possible environmental effect need to be carefully considered. Solvothermal techniques are a type of wet chemical synthesis process that is based on the crystallization of substances and should be carried out in a reactor. In this procedure, an organic solvent should be dissolved. Temperature, pH, and reactant concentration all have an effect on the product’s morphology and particle size. This is a cheap method for producing high-purity, high-quality nanoparticles at low temperatures.

In order to explore the use of HEM for electrochemical water splitting, Jiang et al. synthesized a high-entropy metal hydroxymethoxy-based material FeCoNiMgCr(OH)(OCH_3_), also known as FeCoNiMgCr-HM, using a one-step solvothermal technique. The exceptional catalytic capabilities of electrochemically activated FeCoNiMgCr-HM were a result of the methoxy-induced surface reformation and the combined action of the five metal cations. A simple solvothermal technique developed by Broge et al. produces phase-pure homogeneous HEA nanoparticles made up of eight distinct elements: Pt, Ir, Pd, Rh, Ru, Cu, Ni, and Co [126].

### Mechanical alloy

Mechanical alloying synthesis involves the repeated cold welding, fracturing, and rewelding of powder particles in a high-energy ball mill. Through these mechanical actions, the elements in the powder mixture are homogeneously mixed at the atomic level, resulting in the formation of high-entropy alloys. The high-energy ball milling method is employed to achieve fine particle sizes, enhance mixing, and promote uniformity in high-entropy material synthesis. The repeated impact and deformation of powder particles lead to alloying and the formation of a high-entropy solid solution. Researchers and engineers need to carefully consider factors such as contamination, energy consumption, and processing time to optimize the technique for specific alloy compositions and applications.

Rekha et al. reported employing mechanical ball-milling and ultrasound-assisted exfoliation techniques to synthesize multi-component NiFeCrCoCu HEA nanoparticle-graphene composites [127]. FeNiCoCrAlMn HEA with scattered alumina was synthesized by Prasad et al. by mechanized synthesis. The findings indicate that the alloy’s organization was unaffected by the alumina dispersion. Both alloys exhibit biphasic structures, FCC and BCC, with a minor amount of Mn_3_Co_7_ phases [128].

### Carbothermal shock method (CTS)

One of the easiest synthetic techniques for creating single to polyelemental metal nanoparticles (NPs) is carbothermal shock (CTS). This approach involves putting a metal precursor onto a carbon substrate, raising the temperature instantly by delivering an electric current through the sample for a brief period of time, and producing NPs [19]. The method readily achieves well-dispersed and ultrasmall nanoparticle sizes owing to the short heating/quenching time scales, typically ranging from milliseconds to seconds. Consequently, the CTS method yields non-agglomerated nanoparticles with optimal regulation over particle size, structure, and elemental composition. While the carbothermal shock method presents benefits such as quick synthesis, uniform mixing, energy efficiency, and adaptability, it is essential to take into account challenges concerning scalability, the complexity of equipment, sensitivity of precursors, and control parameters when assessing its suitability for particular applications in high-entropy material synthesis.

Xie et al. used the carbothermal shock method to quickly heat and cool metal precursors on oxygenated carbon carriers in order to create CoMoFeNiCu HEA NPs. Furthermore, the bimetallic CoMo alloy’s mixed-phase restriction was overcome by varying the Co/Mo element ratio in the HEA NPs [129]. Yao et al. used a flash heating and cooling technique to successfully support eight elemental HEA-NPs on conductive activated carbon fiber using the carbothermal shock method [20].

### Spray pyrolysis

The spray pyrolysis method deposits thin films or coatings onto a substrate by atomizing a precursor solution into fine droplets, followed by high-temperature pyrolysis. It is frequently used for synthesizing various materials, including high entropy materials (HEMs). For some applications, the spray pyrolysis process is flexible due to its benefits, including thin film production and homogenous coating. It is important to carefully analyze the limitations of this synthesis process for high entropy materials, including its applicability for thin films and possible issues with precursor breakdown, energy consumption, and equipment complexity. Using the flame spray pyrolysis (FSP) approach, Phakatkar et al. created the unique quinary HEO NPs, which are made up of oxide forms of the normally immiscible elements copper (Cu), manganese (Mn), iron (Fe), nickel (Ni), and zinc (Zn) [130]. On single-crystal sapphire Al2O3 and amorphous SiO2 substrates, Kamecki et al. investigate the low-temperature spray-pyrolysis fabrication of HEO thin film and the determination of its basic properties as well as results concerning the structural and electrical properties of the (Mn,Co,Fe,Ni,Cr)_3_O_4_ composition [131].

### Laser cladding

The laser-related synthesis of high-entropy materials involves using laser irradiation to induce reactions among precursor materials. The focused and intense energy from the laser facilitates the melting, mixing, and solidification of components, resulting in the production of high-entropy alloys [132–134]. Laser-related synthesis of high-entropy materials provides benefits like precision, speed, limited heat impact, and potential for complex shapes. Yet, challenges in equipment complexity, sample size restrictions, precursor material limitations, and safety concerns must be addressed when assessing its suitability for specific applications.

A laser is used to melt and fuse a layer of precursor materials for high-entropy alloys onto a substrate in the laser-related synthesis of high-entropy materials through laser cladding. As a result, a clad layer forms and gives the substrate’s surface high-entropy material characteristics.

Novel high-temperature materials were prepared by Zhang et al. by laser cladding, including a new refractory HEA coating with an equiatomic composition of TiZrNbWMo. The investigation focused on the coating’s hardness and microstructure both prior to and during high-temperature annealing [135]. Ye and colleagues have effectively created high-entropy alloys Al_X_ FeCoNiCuCr in situ by means of laser cladding. Their aim was to create alloy coatings that exhibit favorable combination properties, with a particular emphasis on high-temperature hardness [136].

### Magnetron sputtering

Magnetron sputtering is a technique that deposits thin films or coatings onto a substrate by ionizing and sputtering target materials [137]. In the synthesis of high entropy materials (HEMs), this method utilizes multiple target materials with the desired elemental constituents. The atoms sputtered from these targets are subsequently deposited onto a substrate, forming a thin film with high entropy characteristics. Magnetron sputtering provides benefits like precise composition control, uniform coating, and high purity in synthesizing high entropy materials. However, challenges, including complex equipment, restrictions to thin film applications, and energy consumption, necessitate careful consideration when selecting this method for HEM synthesis.

High entropy films (HEFs) with non-equimolar stoichiometry of Fe33Co30Ni16Al7Mn9W5 were created by magnetron sputtering from mosaic targets at temperatures of 20, 200, 400, and 600 degrees Celsius, respectively. Kim et al. used a reactive direct current magnetron sputtering technique with a TiZrHfNiCuCo HEA powder target to create metallic and nitride coatings on tungsten carbide substrates. He explored how sputtering parameters (power, duration, and N2 reactive gas) affect the microstructure and mechanical properties (hardness and elastic modulus) of HEA metallic and nitride coatings [62].

### Combustion

In contrast to traditional sintering approaches, combustion synthesis stands out as a cost-efficient and straightforward preparation method. The combustion synthesis of high-entropy materials involves a self-sustained exothermic reaction under high gravity conditions, leading to a substantial reduction in processing time. Despite its demonstrated effectiveness for various ceramic materials, the utilization of this method in the context of high-entropy alloys (HEAs) has been relatively constrained. This process initiates a rapid combustion reaction among precursor materials, leading to the formation of high-entropy alloys. Combustion synthesis of high-entropy materials provides cost-effectiveness, speed, simplicity, and potential scalability benefits. Yet, challenges in controlling microstructure, managing precursor reactivity, ensuring material homogeneity, and dealing with limitations in applicable compositions need consideration for specific synthesis applications.

Li et al. studied the microstructures and mechanical properties of AlxCoCrFeNi (*x* = 0, 0.3, 0.5, 0.75, and 1) HEAs synthesized under high gravity. As x shifted from 0 to 1, the structure of AlxCoCrFeNi transitioned from single FCC to single BCC phases [138]. The study by Kuibao Zhang and colleagues. It was possible to produce a unique high-entropy transparent ceramic with a mixed pyrochlore/deficient fluorite structure (La_0.2_Nd_0.2_Sm_0.2_Gd_0.2_Yb_0.2_)_2_Zr_2_O_7_. Investigations were done on the synthesis process, crystal structure, microstructure, element distribution, and optical property [139].

### Microwave-assisted synthesis

The synthesis of high-entropy materials through microwave assistance utilizes microwave radiation for rapid and uniform heating of precursor materials. This approach capitalizes on the efficient coupling of microwaves with specific materials [140–143], resulting in increased heating rates and the facilitation of high-entropy alloy synthesis. The swift and targeted heating within a microwave field allows for the creation of distinctive material structures endowed with desirable properties. Utilizing microwaves for high-entropy material synthesis presents benefits like quick and even heating, energy efficiency, improved purity, and the capacity for customizing properties. Nevertheless, one must consider challenges concerning equipment complexity, scalability, material compatibility, and control factors when assessing its suitability for specific applications.

According to Veronesi et al.’s study, high entropy alloys FeCoNiCuAl, FeCrNiTiAl, and FeCoCrNiAl_2.5_ have been created through the use of microwave-assisted combustion synthesis, which is ignited and maintained in regions of dominant electric or magnetic field in rectangular single-mode applicators that operate between 2450 and 5800 MHz [144]. In a work by Kheradmandfard et al., HEO (Mg, Cu, Ni, Co, Zn)O nanoparticles were created for the first time using microwave irradiation utilizing an ultrafast, highly efficient, low-temperature green approach. With a remarkable reversible capacity of 400 mAh/g at a current density of 0.100 A/g and more than 250 mAh/g at a current density of 5 A/g, the synthesized HEO nanoparticle electrode demonstrated exceptional lithium storage capabilities when employed as anode materials for Li-ion batteries. A remarkable capacity retention of over 98% was achieved at 1 A/g after 1000 cycles [145].

### Plasma sintering

Utilizing plasma, a state of matter composed of charged particles, is integral to the synthesis of high-entropy materials (HEMs). Within the energetic environment of plasma, precursor materials undergo ionization, resulting in the synthesis of high-entropy alloys. The controlled conditions and elevated temperatures facilitate the creation of distinct material structures endowed with enhanced properties. It helps in achieving rapid and uniform densification of high-entropy powders, resulting in a dense and homogeneous material. In the study by Toroghinejad et al., FeCrCoNiCu HEA was created using SPS and MA procedures. At various phases, the alloy’s mechanical, microstructural, and structural qualities were assessed [146]. A study by Yang et al. investigated the cost-effective enhancement of corrosion and tribological performance by adding silicon to the FeCoCrNiMo high-entropy alloy system. The FeCoCrNiMoSi_x_ (*x* = 0.5, 1.0, 1.5) alloys were manufactured using spark plasma sintering (SPS) in order to produce totally dense samples. Including high-temperature oxidation and water corrosion, the study methodically examined microstructure, hardness, tribological characteristics, and corrosion resistance. Durability, hardness, tribological behavior, and persistent anti-oxidation capabilities at elevated temperatures were significantly improved with the addition of silicon [147]. The problem for the plasma sintering will be for preparing samples with large sizes.

### Electrochemical deposition

Electrodeposition serves as a highly efficient method for the fabrication of metal nanoparticles (NPs) [148–153]. It allows for precise control over the nucleation and growth of NPs with diverse morphologies, and the electrolytic deposition process itself is straightforward. Moreover, adjustments to compositions can be easily achieved by altering the relevant parameters. During electrodeposition, metal ions from a solution are reduced and deposited onto an electrode surface, leading to the formation of a high-entropy alloy. While electrodeposition provides benefits in terms of controlling composition, adjustability, and conformal coating for high-entropy materials synthesis, it is accompanied by challenges such as constraints related to substrates, intricacies in multicomponent systems, stability issues in precursor solutions, and energy consumption. The selection of a synthesis method should be based on the specific needs of the desired high-entropy material and its intended application.

According to Aliyu et al.**,** mild steel substrates were electrodeposited with equi-atomic AlCrFeCoNiCu HEA-GO composite coatings. The study examined the microstructural alterations and electrochemical efficacy of electrodeposited AlCrFeCoNiCu HEA, both with and without GO composite coatings, in a 3.5 wt% NaCl solution [154]. Electrochemical separation from a nonaqueous solution has been effectively used to manufacture AlCoCrFeNi multicomponent alloy-based thin films, as reported by Kemény et al. The SEM–EDX method’s composition investigations corroborate this as well [155].

## Applications

### Biomedical applications

HEMs have also attracted significant attention in biomedical field due to their biocompatibility, corrosion resistance, and mechanical strength. Desired property for biomaterials is to have low modulus of elasticity to avoid stress shielding, maintaining high yield strength, fatigue resistance, and high ductility to handle loads from physical activity [156]. HEMs are meeting all of the criteria. It is imperative to explore novel metallic materials that have good biomechanical compatibility such as low Young’s modulus and good strength/ductility balance. Routine issue during diagnosis for loose implant or premature failure is their manetic susceptibility. Low magnetic susceptibility through innovative routes such as HEMs is urgently required which will increase practical applications as biomedical components.

#### Implants and prosthetics

Traditional titanium alloys, such as Ti-6Al4V, confront issues such as stress shielding and the potential release of harmful ions. Motallebzadeh et al. [157] found that Ti_1.5_ZrTa_0.5_Hf_0.5_Nb_0.5_ HEA has much thicker corrosion-resistant barrier oxide coatings, with improved pitting and general corrosion resistance in physiological conditions.

Ishimoto et al. [158] created Ti_1.4_Nb_0.6_Ta_0.6_Zr_1.4_Mo_0.6_ via selective laser melting, obtaining a yield strength of 1690 ± 78 MPa and Young’s modulus of 140 ± 9 GPa. Yuan et al. [159] found that Ti_35_Zr_35_Nb_25_Ta_5_ has improved properties, including a yield strength of 1050 MPa and a substantially reduced Young’s modulus of 69 GPa, which better matches natural bone parameters compared to famous Ti-6Al-4 V alloy which suffers from a large degree of biomechanical incompatibility due to their relatively high elastic modulus about 90 GPa higher than that of human bones and release of toxic ions such as V and Al that can impact nervous system. These studies show HEMs can better match natural bone parameters and can lead to higher wear resistance, long-term stability, and low magnetic susceptibility through microstructural optimization in biomedical HEAs without compromising biocompatibility. These properties are ideal for hip and knee replacements that require high fatigue resistance, dental implants needing excellent osseointegration, spinal implants where stress distribution is critical, and long-term implants requiring superior corrosion resistance.

#### Bone tissue engineering

The TiTaHfNb HEA system, created by Gurel et al. [160], with the compositions that have exceptional bone tissue engineering properties, attaining a Young’s modulus of 112.2 GPa and improved energy absorption through regulated dislocation activity.

**Fig. 6:**
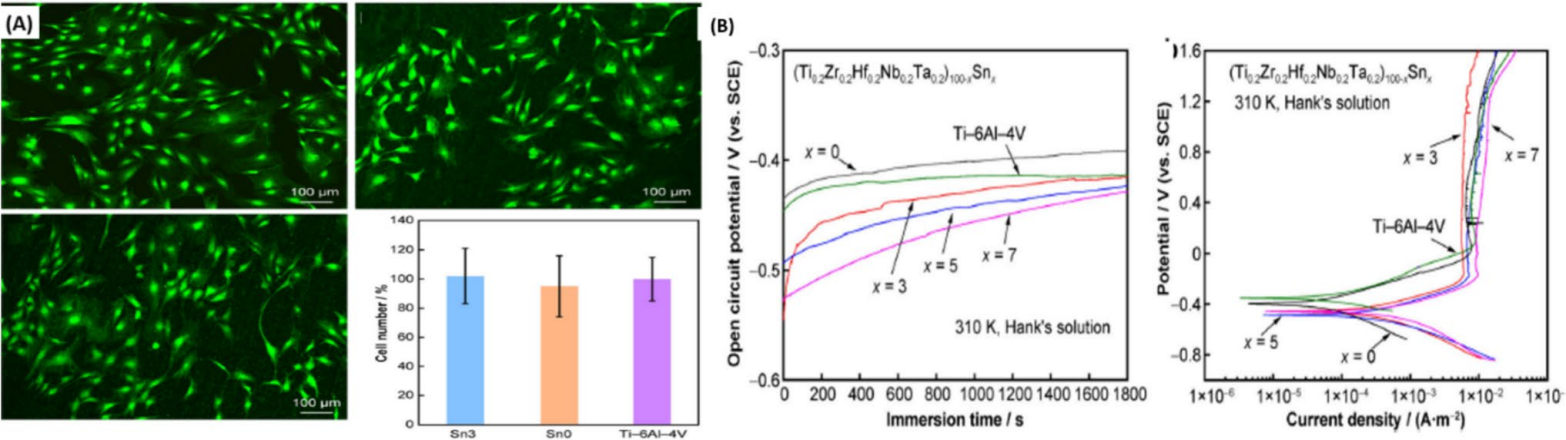
**a** Shows biological performance through cell viability and adhesion. Live/dead staining images of MC3T3-E1 cells on Sn3 HEA, Sn0 HEA, and Ti-6Al-4 V alloy with quantitative comparison. **B** Shows corrosion resistance by the open circuit potential changes over time and potentiodynamic-polarization curves [161]

Yang et al. [161] showed that TiZrHfNbTa-based high-entropy alloys offer favorable biological performance with MC3T3-E1 cells, displaying great cell viability and cell adhesion with statistically no difference to Ti-6Al-4 V (Fig. 6A). The system has commendable corrosion resistance in Hank’s solution, with corrosion rates approximately 10^ − 4 mm/year (varying from 5.6 × 10^ − 4 to 8.2 × 10^ − 4 mm/year based on Sn concentration), comparable to that of Ti-6Al-4 V. All alloys exhibited spontaneous passivation with passive current densities akin to Ti-6Al-4 V (Fig. 6B).

#### Cardiovascular applications

High-entropy materials exhibit remarkable potential in cardiovascular applications, especially in the production of stents. Akmal et al. [162] established that the (MoTa)xNbTiZr system at *x* = 0.2 has enhanced corrosion resistance, attaining passivity values of 1.2 V without pitting under physiological settings, markedly surpassing conventional 316L stainless steel stents. Kartik et al. [163] explore the mechanical behavior and microstructure of Al_0.1_CoCrFeNi HEA subjected to thermo-mechanical processing and its potential application in peripheral vascular stent implants that are prone to high failure rates. The result demonstrated HEM alloy possesses characteristics that compare well against currently used stent materials, and it can potentially find use in peripheral vascular stent implants and extend their life cycle. These materials exhibit enhanced mechanical flexibility, crucial for dynamic cardiovascular settings, while preserving structural integrity and biocompatibility. Along with enhanced corrosion resistance and fatigue resistance, HEMs can be explored for properties such as superior endothelial cell response, hemodynamic performance, thrombogenicity, and endothelialization. This will make HEMs suitable for coronary stents that require excellent fatigue resistance, heart valves needing superior hemodynamic performance, blood-contacting devices requiring minimal thrombogenicity, vascular grafts requiring enhanced endothelialization, and any cardiovascular implants that require minimal inflammatory reaction and prolonged stability.

### Electronics and devices

High-entropy materials (HEMs) have demonstrated promising results in practical applications, particularly microwave absorption and electromagnetic interference (EMI) shielding. These applications are becoming increasingly significant as the number of electronic gadgets, wireless communication systems, and defense technologies that require good electromagnetic wave management grows.

#### Sensor and actuators

Conventional sensor and actuator materials have substantial difficulties in attaining high sensitivity while ensuring stability over extensive temperature ranges and under adverse conditions.

**Fig. 7:**
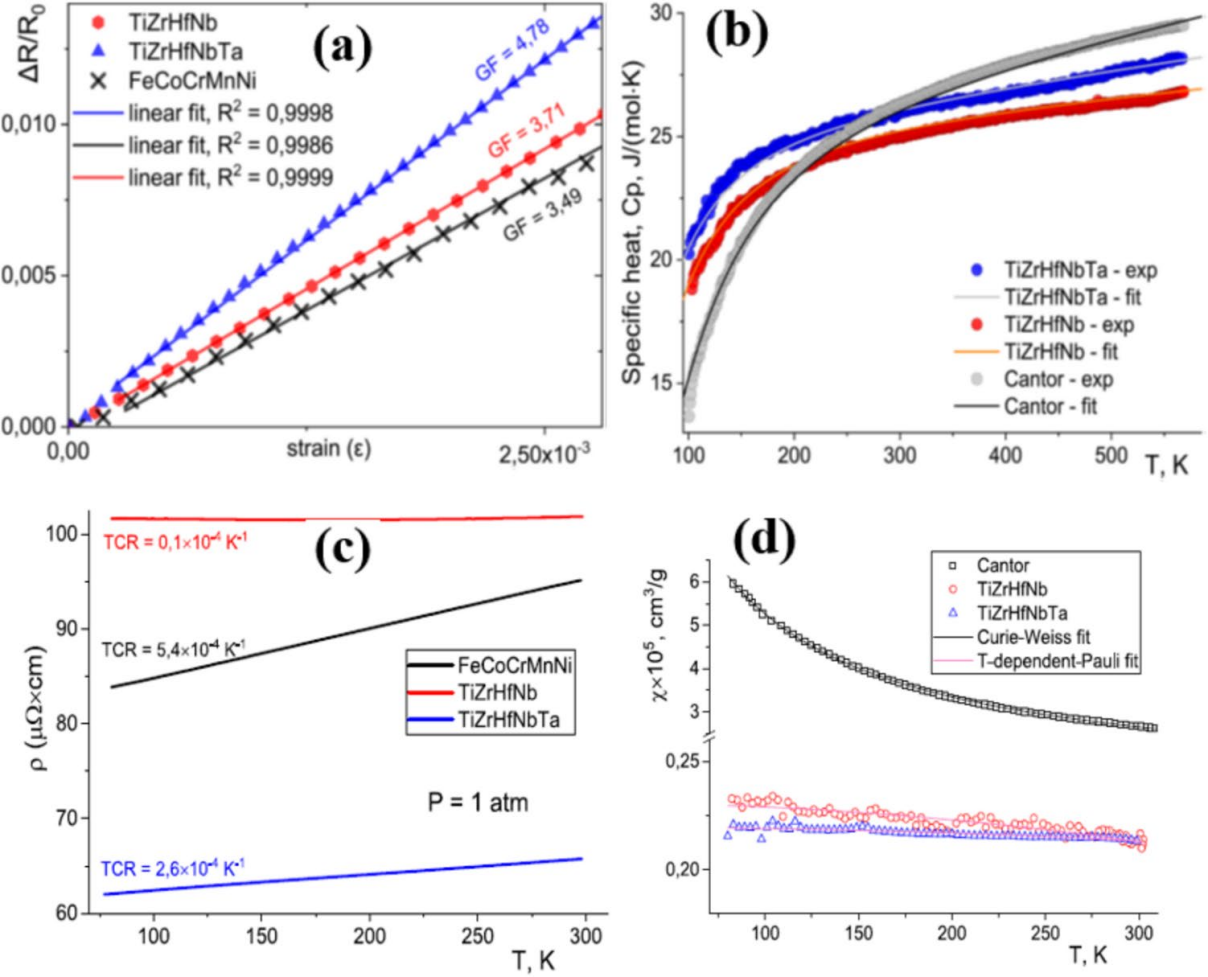
**a** Relative resistivity ΔR/R0 vs. strain ε for the cold-rolled HEAs, measured at ambient temperature and pressure. **b** Specific heat of the as-cast HEAs vs. temperature. **c** Electrical resistivity of the as-cast HEAs vs. temperature at ambient pressure. **d** Magnetic susceptibility of the as-cast HEAs vs. temperature at ambient pressure [164]

Uporov et al. [164] establish that HEAs such as Cantor alloy (TiZrHfNb, TiZrHfNbTa, and FeCoCrMnNi) and its derivatives as well as refractory ones exhibit constant resistivity over a large temperature span. This property is extremely important for an alloy to be used as an electrical sensor or resistor. TiZrHfNbTa high-entropy alloy-based strain sensors attain remarkable gauge factors from 3.49 to 4.78, significantly exceeding the performance of conventional metal strain gauge such as constantan (GF 2–2.2) and manganin (GF 0.5–0.7). HEMs showed temperature stability over temperature range of − 173 °C to 247 °C (100–500 K) and showed no abnormal behavior. Electrical and magnetic stability over experience temperature range of − 196 °C to 27 °C showed a weak temperature dependence of electrical resistance in these ranges, and no magnetic ordering was observed down to − 196 °C weak time independent drift. These properties are attributed defective crystal structure of high-entropy alloys resulting in combination of properties such as showing single phase solid solution, microstructure showing finest dendrites, and cold-roll showing texturing and twinning effects. The dendritic microstructure and texturing effects from cold-rolling influence their mechanical properties, and the irregular atomic arrangements affect their electronic and thermal transport properties. These properties make HEMs particularly suitable for sensing and actuation applications such as high-temperature environments, applications that require long-term stability, harsh environment operation, and multi-functional sensing platforms and can find strong foothold in nuclear reactor monitoring, aerospace sensing, deep well analysis, etc., where conventional sensors have limitations. The typical signals are displayed in Fig. 7.

#### Electromagnetic wave absorption

**Fig. 8:**
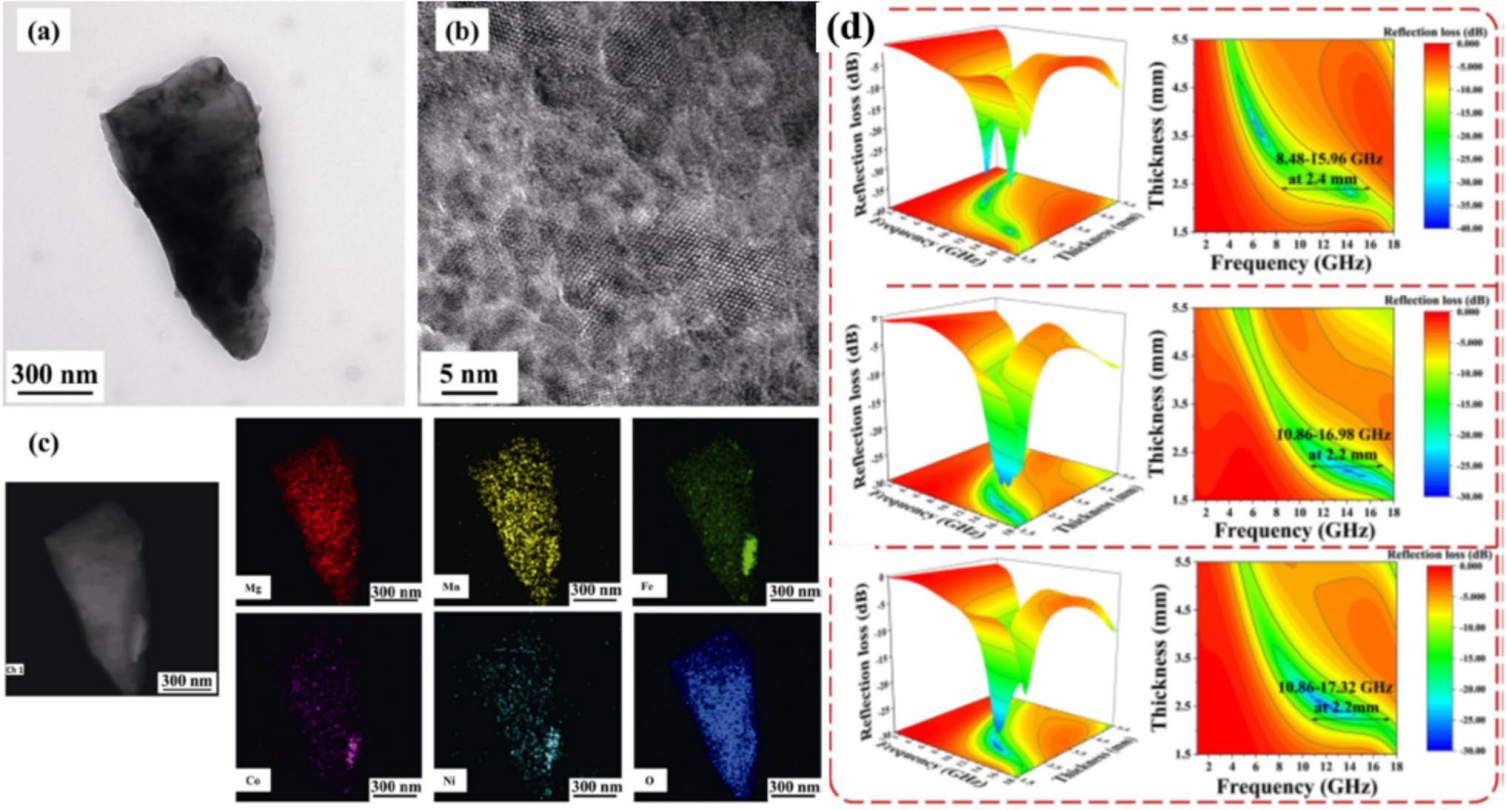
**a** TEM image shows nano-domain structure within (Mg_0.2_Mn_0.2_Fe_0.2_Co_0.2_Ni_0.2_)Fe_2_O_4_ grain. **b** HRTEM image. **c** Scanning transmission electron microscopy (STEM) image and EDS mappings of (Mg0.2Mn0.2Fe0.2Co0.2Ni0.2)Fe2O4. **d** 3D reflection loss plot demonstrating broad absorption bandwidth [113]

High-entropy spinel ferrites (HEF) exhibit remarkable electromagnetic wave absorption properties due to their distinctive microstructural characteristics. Ma et al. [43] demonstrated that (Mg_0.2_Mn_0.2_Fe_0.2_Co_0.2_Ni_0.2_)Fe_2_O_4_ attains a minimal reflection loss of − 35.10 dB at 6.78 GHz (Fig. 8d), which is ascribed to its nano-domain architecture inside grains, as evidenced by TEM examination (Fig. 8a–c).

The nano-domains amplify interfacial polarization effects in electromagnetic fields, while the significantly disordered cation distribution between tetrahedral and octahedral sites was evidenced by XPS with Fe3 + (tet):Fe3 + (oct) ratios of 1:2.06. This elevates defect concentration and ionic conductivity, resulting in enhanced dielectric properties. The hopping mechanism between Fe^2+^ and Fe^3+^ ions, enabled by oxygen vacancies generated by the high-entropy effect, further amplifies the dielectric loss capacity. Mohammadabadi et al. [165] demonstrated that the integration of graphene into (MnNiCuZn)_0.7_Co_0.3_Fe_2_O_4_ generates supplementary interfaces among HEF/paraffin, HEF/(graphene + paraffin), and (HEF + graphene)/paraffin, hence enhancing interfacial polarization losses. The combined loss mechanisms—magnetic loss due to ferromagnetic resonance (shown by permeability peaks at 4–12 GHz) and increased dielectric loss from nano-domains and interfaces—yield an effective absorption bandwidth of 7.48 GHz. The saturation magnetization and coercivity can be adjusted via composition, with (Mg0.2Mn0.2Fe0.2Co0.2Ni0.2)Fe2O4 demonstrating the highest saturation magnetization of 56.10 emu/g owing to optimal distribution of Fe-group ions, whereas (Mg_0.2_Mn_0.2_Fe_0.2_Co_0.2_Ni_0.2_Cu_0.2_)Fe_2_O_4_ displays the greatest coercivity of 204.52 Oe attributable to its reduced grain size. The octahedral grain shape, averaging 2–3 μm, regulated by the relative growth rates of {111} and {200} crystal faces, offers an ideal particle size for optimizing magnetic and dielectric properties. These multi-component systems exhibit enhanced electromagnetic absorption relative to traditional ferrites due to their synergistic dielectric-magnetic loss mechanisms facilitated by their intricate microstructure.

#### Energy conversion and storage

With the increasing awareness of fossil energy and environmental pollution [166–169], clean energy such as hydrogen, fuel cells [170–175], and capacitors [176–180] has attracted much attentions. High entropy hydroxides have been prepared and served as electrodes for supercapacitors. The energy density of the prepared supercapacitors is highly promoted [181]. The researchers have demonstrated that the doped metallic elements decreased the band gap, changed the density state at the Fermi level, and increased the material conductivity. In addition, the charge density and Mulliken charge analysis reflected the electron transfer between high entropy-LDH metals. This accelerated the electron/ion transfer rate in electrochemical reactions and caused an enhanced electrochemical performance. This demonstrated the energy applications of the high-entropy compounds. However, how to control the shape is a challenge and should be exploited for the future.

## Conclusion and perspectives

High-entropy materials (HEMs) being an innovative category of advanced materials that utilize configurational entropy for phase stabilization have resulted in remarkable features for many applications. With exceptional control over composition and microstructure through diverse synthesis methods including as arc melting, powder metallurgy, solvothermal synthesis, and carbothermal shock techniques, the incorporation of rare-earth elements demonstrates a significant potential in adjusting magnetic characteristics, with La-based compounds displaying a spontaneous magnetization of approximately 15.2 emu/g and consistent alterations in magnetic ordering temperatures. The capacity to modify composition for tunability signifies substantial progress in materials design and facilitates customized features for particular applications.

Biomedical applications have demonstrated considerable potential, especially in implant materials, where TiZrHfNbTa-based systems exhibit superior cell and mechanical compatibility (Young’s modulus ~ 69 GPa) and higher corrosion resistance relative to traditional materials. The decreased elastic modulus more closely aligns with natural bone characteristics while preserving substantial strength and superior biocompatibility. In cardiovascular applications, high-entropy materials such as (MoTa)xNbTiZr exhibit exceptional corrosion resistance, with passivity values of 1.2 V under physiological conditions, exceeding those of conventional stainless steel implants.

In electromagnetic applications, high-entropy spinel ferrites exhibit remarkable microwave absorption, achieving a reflection loss of − 35.10 dB at 6.78 GHz, due to their nano-domain structure and combined dielectric-magnetic loss processes. The integration of several components in regulated ratios has facilitated unparalleled management of electromagnetic characteristics, applicable in fields such as telecommunications and stealth technologies. High-entropy oxides and ceramics demonstrate considerable potential in attaining substantial dielectric and magnetic losses essential for efficient microwave absorption across a wide frequency range.

The advancement of innovative synthesis processes and characterization methodologies has been crucial in enhancing our comprehension of these materials. Computational methodologies, such as CALPHAD and machine learning, have arisen as effective instruments for forecasting phase stability and directing alloy design, markedly expediting the identification of novel compositions. Nonetheless, other obstacles persist, such as the scalability of synthesis techniques, evaluation of long-term stability, and economically viable production. The intricate connections among various constituents and their impact on characteristics necessitate additional exploration using advanced characterization techniques.

The domain of HEMs is poised for expansion, showcasing significant advancements in materials design, processing, and applications. Future studies should concentrate on utilizing artificial intelligence and machine learning for composition prediction, establishing uniform testing methodologies, and investigating innovative element combinations for improved functionality. The incorporation of sustainable practices in synthesis and processing will gain significance, coupled with the advancement of recycling strategies for these intricate materials. The ongoing development of high-entropy materials, facilitated by sophisticated characterization methods and computational resources, is poised to reveal new opportunities in next-generation applications, including biomedical implants and electromagnetic interference shielding, establishing HEMs as a vital class of materials for future technological progress.

## Data Availability

No datasets were generated or analysed during the current study.
